# Use of Meropenem and Other Antimicrobial Lock Therapy in the Treatment of Catheter-Related Blood Stream Infections in Neonates: A Retrospective Study

**DOI:** 10.3390/children9050614

**Published:** 2022-04-26

**Authors:** Fiammetta Piersigilli, Cinzia Auriti, Andrea Dotta, Bianca Maria Goffredo, Sara Cairoli, Immacolata Savarese, Francesca Campi, Tiziana Corsetti, Iliana Bersani

**Affiliations:** 1 Department of Neonatology, Cliniques Universitaires Saint Luc, Université Catholique de Louvain, 1200 Bruxelles, Belgium; 2 Department of Medical and Surgical Neonatology, Bambino Gesù Children’s Hospital, 00165 Rome, Italy; cinzia.auriti@opbg.net (C.A.); andrea.dotta@opbg.net (A.D.); immacolata.savarese@opbg.net (I.S.); francesca.campi@opbg.net (F.C.); iliana.corsetti@opbg.net (I.B.); 3 Biochemistry Laboratory, Department of Specialist Pediatrics, Bambino Gesù Children’s Hospital, 00165 Rome, Italy; biancamaria.goffredo@opbg.net (B.M.G.); sara.cairoli@opbg.net (S.C.); 4 Unit of Clinical Pharmacy, Bambino Gesù Children’s Hospital, 00165 Rome, Italy; tiziana.corsetti@opbg.net

**Keywords:** central venous catheter, CLABSI, CRBSI, bloodstream infection, lock therapy, NICU, neonate

## Abstract

(1) Background: Newborns admitted to Neonatal Intensive Care Units (NICUs) often require the placement of central vascular catheters (CVC), which are a major risk factor for hospital infection. Numerous strategies exist to prevent central line-associated blood stream infections (CLABSIs) and catheter-related bloodstream infections (CRBSIs), with only a few offering options to save the catheter when it is impossible to replace. CRBSIs continue to be a major problem for neonates in NICUs. Most CRBSIs are resistant to systemic antibiotics due to the presence of intraluminal bacterial biofilm. Therefore, catheter removal is frequently necessary when a CRBSI occurs. The so-called Antibiotic Lock Therapy (ALT) is an antimicrobial therapeutic strategy which seems to be promising in neonates when catheter removal is difficult due to critical conditions. To date, evidence about the use of ALT in the neonatal period is still fragmentary, since only poor and heterogeneous data exist. (2) Methods: We report our successful experience with ALT in seriously ill neonates with CRBSI for whom the replacement of the catheter could have been life threatening. (3) Results: ALT repetitively performed for at least 12 h was effective in 11 out of 13 infants (84.6%). It was not effective in two infants in whom ALT was performed for only 6 h. Moreover, we present new data about the stability testing of meropenem for its use during ALT in neonates. (4) Conclusions: When CRBSI occurs—bearing in mind that the optimal management is catheter removal if antibiotic therapy is not effective within 48 h—ALT seems to be a valid alternative therapy when removal is impractical due to critical conditions.

## 1. Introduction

Newborns admitted to Neonatal Intensive Care Units (NICUs) often require the placement of Central Vascular Catheters (CVCs) to allow the administration of fluids, drugs, parenteral nutrition and monitoring. However, despite the advantages of CVCs, the creation of multiple entryways through the skin barrier, both along the external surface of the catheter and through the lumen, increase the occurrence of infectious complications. Catheter-associated bloodstream infections (CLABSIs) and catheter-related bloodstream infections (CRBSIs) are the most prevalent issues [1].

Bacterial infections can be cleared by both the host immune system (such as opsonization and phagocytosis) and treatment with systemic antibiotics. However, bacteria may bind to the internal surface of the catheter and form biofilms where the cells are effectively protected by self-secreted extracellular polymeric substances (EPSs). The mechanisms by which this biofilm protects bacteria against natural immunity may consist of: limited penetration of leukocytes into the biofilm; decreased capacity of the leukocytes to phagocytize bacteria imbedded in the biofilm; the inactivation of leukocyte-specific processes by the biofilm matrix (as secretion of the cytokine IFN-γ); and genetic switches leading to increased resistance to components of the human immune system.

In this context, bacterial susceptibility to conventional antimicrobial therapy is significantly lower in biofilms compared to their planktonic microbial counterparts.

Therefore, when a CRBSI is diagnosed in a patient and blood culture is still positive despite 48 h of systemic antibiotic treatment, the removal of the CVC is strongly recommended, in particular, when infections are caused by *Staphylococcus epidermidis*, *Streptococcus viridans*, *Pseudomonas aeruginosa*, *Klebsiella pneumoniae*, *Enterococcus* spp. or *Candida* species, due to their particular ability to form intraluminal biofilm [2,3]. In these cases, if the neonate still requires a central line, a new catheter should be placed, avoiding the previous site of insertion [4].

Nevertheless, the placement of a new CVC might be challenging in cases of extremely sick and/or preterm neonates. If patients’ conditions are too critical to undergo catheter removal, or if they cannot face the placement of a new central line, as is recommended, a possible therapeutic option besides the administration of systemic antimicrobial drugs is the so-called Antibiotic Lock Therapy (ALT) [5].

ALT, i.e., a local administration of high-concentration antibiotics into the catheter lumen, is a possible option to achieve catheter salvage, providing effective antibiotic concentrations at the site of infection [6,7]. To date, only poor and fragmentary evidence exists about this therapy in neonates, especially concerning the optimal drug dose and the timing.

The present paper summarizes our experience with the use of ALT in neonates. Moreover, it provides new data about the stability testing of meropenem for its use during ALT in neonates, which, to our knowledge, are not available in the literature yet.

## 2. Patients and Methods

We retrospectively reviewed all cases of CLABSI and CRBSI from March 2015 to March 2019 at the Department of Medical and Surgical Neonatology at the Bambino Gesù Children’s Hospital, Rome. We excluded central venous umbilical catheters from the analysis. As a NICU and neonatal surgical unit, about 80% of the newborns hospitalized in this unit have a central vascular catheter, positioned for medical treatments and parenteral nutrition. In the unit, we have a strict CVC insertion and maintenance routine. This involves adequate staff training, maintaining accurate reporting schedules for CVC care, chlorhexidine skin disinfection, maximum use of aseptic barriers, no-touch techniques, reducing unnecessary access to the line, the preparation of antibiotic therapies under a sterile hood in the hospital pharmacy, and the removal of the catheter as soon as it is not necessary anymore.

Overall, 600 CVCs were positioned during the study period. Among these, 321 were 1 Fr or 2 Fr epicutaneo cava catheters (ECC), 239 were 2 Fr or 3 Fr ultrasound-guided centrally inserted central catheters (CICC), and 40 were tunneled long-term catheters.

When a CLABSI was suspected (neonates showing less vigorous sucking, apnea, bradycardia, temperature instability, respiratory distress, or abdominal distention), the neonate promptly received empirical systemic antimicrobial treatment and CVC removal was considered if the systemic antibiotics turned out to be ineffective after 72 h. The diagnosis of CRBSI was possible only for CICC and tunneled central catheters, since it is not possible to draw a blood sample for culture from ECCs. ALT is not performed for ECCs, as it is not possible to diagnose a CRBSI or perform a lock in ECC catheters, as they need a continuous infusion of at least 1 mL/h to avoid occlusion. When neonatal conditions did not allow CVC removal, ALT was performed. The antimicrobial drug to be instilled for the ALT into the catheter lumen was chosen according to each specific antibiogram, as soon as it became available. Since no literature evidence exists on meropenem use for ALT in neonates, our laboratory performed a stability test of meropenem for its use in ALT. 

### 2.1. Meropemen Stability Test

We performed stability tests for meropenem at concentrations of 1 mg, 2 mg and 5 mg after drug reconstitution with saline in association with ethanol 70% and heparin. The solutions were stored at room temperature and analyzed using the HPLC method for the dosage of meropenem at the following time intervals: time 0, time 8 h, time 12 h, time 24 h. At time 24 h, the stability tests were also performed at 4 °C. We determined meropenem concentrations using a validated method with HPLC apparatus Agilent Technologies 1200. The samples were analyzed using a Column Kinetex EVO C18 5µ 100 Å 4.6 mm × 150 mm at the temperature of 25 °C with gradient elution. The flow was 0.8 mL/min. Buffer A consisted of 8.9 g Na_2_HPO_4_ and 1000 mL water (pH 7 with orthophosphoric acid). Buffer B consisted of acetonitrile. The effluent was monitored at a UV wavelength of 295 nm. 

### 2.2. ALT Procedure

The drug to be used for ALT, in association with systemic therapy, should be chosen according to the causative organism of sepsis and the minimal inhibitory concentration (MIC) for that drug. The antibiotic concentration of the instilled solution should be 100 to 1000 times higher than the MIC of the germ [8,9]. ALT can be performed only in CICCs and long-term tunneled central catheters, not in ECCs. A number of different antibiotics and different dosages have been proposed for the neonatal period (Table 1).

We performed ALT with a volume equal to the CVC priming volume plus 0.2 mL. ALT was performed for at least 3 consecutive days and, when possible, up to 10 consecutive days. Following each daily drug instillation, the central line had to remain closed for ALT. As CVCs are used not only for drug administration but also for hemodynamic monitoring, in the most critical neonates it was not possible to leave the catheter closed for more than 6 h. In the less critical neonates, the lock time was increased to 12 or 24 h when possible. Aspiration of CVC content (volume equal to the CVC priming volume plus 0.1 mL of blood) was then performed and, after CVC washing with saline, the normal use of the central line was newly allowed up to the subsequent ALT. During the treatment period, we performed serial blood cultures from CVC to monitor ALT efficacy. We added no anticoagulants to the ALT. No cases of mechanical CVC malfunctioning were recorded.

ALT therapy was performed as a standard of care therapy; therefore, no ethical committee approval was needed. Nevertheless, parents were informed that the lock therapy was used as a standard of care and provided verbal informed consent.

## 3. Results

The results of the stability tests performed with meropenem are presented in Table 2, where the percentage decline in concentration over time is highlighted. A concentration drop of up to 15% from the initial concentration was considered acceptable. Overall, these data show that the optimal concentration in terms of stability is 2 mg/mL, without the addition of ethanol or heparin.

During the study period, the incidence of CLABSI in our unit was 1.7/1000 catheter days. Overall, ALT was performed in 13 neonates as an alternative to CVC removal. In order to perform effective ALT, the catheter needs to be locked for a long period of time (at least 12 h). As the neonates receiving ALT were the most critical patients, the time of the lock was decided based on the condition of the neonate. For example, we managed one neonate with a peripheral line during the lock, and then used the catheter to give parenteral nutrition and medications requiring a central line. This is the reason why we did not use a standard lock dwell time, and the most critical neonates only had 6 h of ALT. 

Meropenem ALT was used in four cases, with a concentration of 2 mg/mL in 0.9% saline solution that allowed for CVC salvage. The other antimicrobials used for ALT were vancomycin, amikacin, and micafungin. The antibiotic concentrations used were 3 mg/mL for amikacin and vancomycin, 0.9% for saline solution (without heparin), and 5 mg/L for micafungin. In the latter case, micafungin was associated with ethanol 70%, as previously described [7]. 

Neonates receiving ALT had a tunneled 2.7 Fr silicon long-term catheter in 10 cases and a centrally inserted 3 Fr polyurethane catheter in 3 cases. Blood cultures were positive for *Klebsiella pneumoniae* in six cases, *Candida albicans* in four cases, *Staphylococcus aureus* in two cases, and *Enterococcus faecalis* in one case. Infection resolution after ALT with negative subsequent bloodstream cultures was achieved in 11/13 (84.6%) neonates with CRBSI. CVC removal due to ALT failure was necessary only in two neonates who received the ALT only for 6 h and 3 days due to their critical conditions requiring continuous infusions through the CVC. Table 3 describes the type of ALT that was performed and the outcomes.

## 4. Discussion

Central line-associated blood stream infections (CLABSI) are episodes of sepsis diagnosed when a CVC is in situ, with the infection occurring from 48 h after CVC insertion to 48 h after CVC removal. Catheter-related blood stream infections (CRBSI) have a precise relationship with the catheter. This type of infection is defined with precise microbiological criteria, i.e., the positivity of CVC blood culture developing at least 2 h before the peripheral blood culture, or the detection of the same organism from the peripheral blood and the catheter lumen blood, with three-fold greater colony count in the latter [18,19]. In CRBSI, there is a cause–effect relationship between the catheter and the infection.

The poor efficacy of systemic antibiotics in the context of CRBSI is due to a number of factors, including the difficulty of achieving effective antibiotic concentrations inside the catheter because of the presence of the intraluminal biofilm. EPS represents about 80–90% of biofilm and varies in chemical and physical properties. It is primarily composed of neutral or polyanionic polysaccharides and other biopolymers such as DNA, proteins and teichoic acids [20]. The polysaccharidic matrix effectively protects bacteria against both the host defense system and antibiotics. It also causes complex metabolic, genomic and growth changes leading to bacterial self-adaptation to external stresses and damage [21]. Lock therapy consists of instilling, within the lumen of the colonized catheter, high concentrations of an antibiotic effective on the germ responsible for the infection and closing the catheter for a period of time [22,23]. Current guidelines on the management of CRBSIs recommend catheter removal if the blood culture is still positive for a fungal infection despite 48 h of systemic antifungal therapy [3], or removal if the blood culture is still positive for a bacterial infection despite 72 h of systemic antibiotic therapy [24]. Consequently, the literature lacks data describing the use of this adjunctive local therapy in the course of sepsis. A recent Cochrane review [25] about the use of ALT concluded that, although preventive ALT appeared to be effective in decreasing CRBSI occurrence in the neonatal period, it did not decrease mortality, and evidence is still insufficient to recommend it, considering the limited number of trials and the heterogeneity of antimicrobial drugs administered [26], stressing the need for further investigations. Nevertheless, many patients, due to their critical clinical condition, cannot face catheter replacement. This situation occurs frequently in severely preterm and/or extremely low-birth-weight infants. In this context, the target is to save the catheter during the treatment of an ongoing infection. Our data support the possibility of saving catheters by performing a catheter lock with antibiotics during infection. In our experience, this procedure was not associated with CVC malfunctioning and was successful in 84.6% of infected neonates. However, the available data are limited and confusing in terms of the exact antimicrobial drug, dosage and timing required for the catheter lock. 

Regarding fungal infections, it is widely recommended to remove the catheter in cases of fungal CLABSI [27]. Lock therapy should be considered in cases of fungal catheter infection only if the catheter cannot be removed. Recently, Walraven et al. published a very interesting mini review on the use of ALT on animal models and in humans to resolve fungal catheter-related infections [28]. ALT was most commonly employed for 14 days in conjunction with systemic antifungal therapy and was stopped after the last negative blood culture. Drug lock concentrations varied: for Amphotericin B, the concentration ranged from 0.33 mg/mL to 2.67 mg/mL, Caspofungin was used in three pediatric patients at a dose of 3.33 mg/mL, and other patients were treated with a 70% ethanol lock, in conjunction with systemic antifungal therapy. Ethanol locks can also be considered in cases of fungal CRBSI [29]. The overall clinical catheter salvage rate was reported was 77%. Echinocandins can also be used for ALT [30]. Our group has also previously reported the successful use of lock therapy with micafungin in association with 70% ethanol to save catheters during fungal infection [7]. 

To our knowledge, no data exist about the use of meropenem as a catheter lock solution. Meropenem is a broad-spectrum antibiotic with excellent activity against some pathogens causing late-onset sepsis (*Escherichia coli*, *Klebsiella* spp., *Enterobacter* spp. and *Pseudomonas* spp.). Our results confirmed a good meropemen stability even after 24 h from the preparation of the lock solution, suggesting its possible use for ALT in neonates.

Our experience in saving catheters was successful and we observed the failure of this procedure only in two patients, in which ALT could be performed only for 6 h and 3 consecutive days. In both cases, the locks were performed with amikacin. The failure we faced was probably due to a combination of the low rate of penetration of the antibiotic into the biofilm and the short lock time we had to use because of the critical conditions of the neonates, which did not allow longer closure of the catheter. 

A more effective ALT therapy has been proposed with taurolidine. Taurolidine has the advantage of covering a broad spectrum of germs and not inducing antibiotic resistance [31]. Until recently, taurolidine could not be used in the pediatric population because of its combination with citrate that contraindicated its use in children. Taurolidine 2% without citrate is now available on the market and is starting to be used in the pediatric population. Its efficacy and safety are being studied in a number of pediatric randomized clinical trials [32,33]. Nevertheless, no studies have been performed on its safety in the neonatal period; therefore, further studies are warranted before its use can be routinely allowed in neonates.

## 5. Conclusions

When CRBSI occurs—bearing in mind that the optimal solution is catheter removal if antibiotic therapy is not effective within 72 h—ALT seems to be a valid alternative therapy when removal is impractical due to critical conditions. The results of our stability tests show that meropenem can be used for ALT. Until further investigations allow the use of taurolidine in the neonatal population, ALT can be effectively used in selected cases of CRBSI in neonates, provided it is instilled for a long enough period.

## Figures and Tables

**Table 1:** Use of different antimicrobial drugs for lock therapy in the neonatal period.

Antimicrobial Drug (Dosage)	Anticoagulant Agent	References
Amikacin (1.0 mg/mL)	No	Domingo [10]
Ampicillin (10.0 mg/mL)	10 or 5000 UI/mL	Robinson [11]
Cefazolin (5.0 mg/mL)	2500 or 5000 UI/mL	Krishnasami [12] Vercaigne [13]
Cefazolin (10.0 mg/mL)	No	Vercaigne [13]
Ceftazidime (0.5 mg/mL)	100 UI/mL	Rijnder [14]
Ciprofloxacin (0.2 mg/mL)	5000 UI/mL	Droste [15]
Gentamicin (1.0 mg/mL)	Heparin 2500 UI/mL	Krishnasami [12]
Gentamicin (5.0 mg/mL)	No	Benoit [16]
Vancomycin (1.0 mg/mL)	Heparin (100 UI/mL)	Robinson [11]
Vancomycin (1.0 mg/mL)	No	Domingo [10]
Vancomycin (2.0 mg/mL)	10 UI/mL	Robinson [11]
Vancomycin (2.5 mg/mL)	2500 or 5000 UI/mL	Krishnasami [12] Robinson [11]
Vancomycin (5.0 mg/mL)	No or 5000 UI/mL	Lee [17] Vercaigne [13]
Vancomycin (2.5 mg/mL) + Gentamicin (1.0 mg/mL)	Heparin 2500 UI/mL	Krishnasami [12]
Cefazolin (5.0 mg/mL) + Gentamicin (1.0 mg/mL)	Heparin 2500 UI/mL	Krishnasami [12]

**Table 2:** Stability testing of meropenem for its use during lock therapy.

1 mg/mL (Room Temperature)	Saline	Ethanol 70%	Heparin
0 h	100%	100%	100%
8 h	89.9%	80.6%	93.6%
12 h	89.5%	77.5%	90.4%
24 h	84.2%	70.5%	78.2%
1 mg/mL (+4 °C)	Saline	Ethanol 70%	Heparin
24 h	93.0%	74.6%	95.1%
2 mg/mL (Room Temperature)	Saline	Ethanol 70%	Heparin
0 h	100%	100%	100%
8 h	99.3%	90.3%	99.9%
12 h	99.2%	83.4%	97.4%
24 h	95.3%	75.2%	92.5%
2 mg/mL (+4 °C)	Saline	Ethanol 70%	Heparin
24 h	94.3%	87.6%	98.1%
5 mg/mL (Room Temperature)	Saline	Ethanol 70%	Heparin
0 h	100%	100%	100%
8 h	98.4%	85.1%	91.9%
12 h	93.7%	80.3%	85.9%
24 h	92.1%	65.9%	84.2%
5 mg/mL (+4 °C)	Saline	Ethanol 70%	Heparin
24 h	93.8%	60.3%	87.9%

**Table 3:** Characteristics of the antibiotic locks performed.

Germ	Type of Catheter	Antibiotic	Dosage	Effective
*Klebsiella pneumoniae*	2.7 Fr long-term tunneled catheter	Meropenem	2 mg/mL in 0.9% saline	Yes
*Klebsiella pneumoniae*	2.7 Fr long-term tunneled catheter	Meropenem	2 mg in 0.9% saline	Yes
*Klebsiella pneumoniae*	2.7 Fr long-term tunneled catheter	Meropenem	2 mg/mL in 0.9% saline	Yes
*Klebsiella pneumoniae*	2.7 Fr long-term tunneled catheter	Amikacin	3 mg/mL in 0.9% saline	No
*Klebsiella pneumoniae*	2.7 Fr long-term tunneled catheter	Amikacin	3 mg/mL in 0.9% saline	No
*Klebsiella pneumoniae*	3 Fr CICC	Meropenem	2 mg/mL in 0.9% saline	Yes
*Enterococcus faecalis*	2.7 Fr long-term tunneled catheter	Amikacin	3 mg/mL in 0.9% saline	Yes
*Staphylococcus aureus*	2.7 Fr long-term tunneled catheter	Vancomycin	3 mg/mL in 0.9% saline	Yes
*Staphylococcus aureus*	3 Fr CICC	Vancomycin	3 mg/mL in 0.9% saline	Yes
*Candida albicans*	2.7 Fr long-term tunneled catheter	Mycamine	5 mg/L 70% ethanol	Yes
*Candida albicans*	2.7 Fr long-term tunneled catheter	Mycamine	5 mg/L 70% ethanol	Yes
*Candida albicans*	2.7 Fr long-term tunneled catheter	Mycamine	5 mg/L 70% ethanol	Yes
*Candida glabrata*	3 Fr CICC	Mycamine	5 mg/L 70% ethanol	Yes
